# HYPERLACTATEMIA IN SEPTIC SHOCK, UNMASKING THE FALSE NOTION; AN INTRIGUING CASE REPORT.

**DOI:** 10.51894/001c.122836

**Published:** 2024-08-30

**Authors:** Mehwish Zeb, Apoorv Tiwari, Mazen Hasan, Abed Dabaja, Sujata Kambhatla

**Affiliations:** 1 Internal Medicine Garden City Hospital

## 18

### INTRODUCTION

Sepsis is defined as a life-threatening dysregulated body response and systemic inflammation to an infection. Septic shock is a subset of sepsis which has cellular, metabolic, and circulatory abnormalities leading to the shift of aerobic respiration to an anaerobic pathway with resultant lactic acidosis. It is a vasodilatory shock and a continuum of severity extending from infection and bacteremia to septic shock. The burden of sepsis and septic shock has consistently increased over the years, affecting millions of people around the globe every year.

### CASE DESCRIPTION

This study illustrates a case of 49 years old female, who presented to the emergency department with intractable back pain, attributed to her caregiver services for her disabled sister. On examination, she was febrile, hypotensive with MAP less than 65 mmHg, cool, mottled skin, and capillary refill was greater than 5 seconds. Based on surviving sepsis campaign guidelines, balanced crystalloid, and empiric antibiotics were initiated immediately. The patient was started on vasopressors because she did not respond to fluid resuscitation and was ultimately transferred to the intensive care unit. Diagnostic studies were carried out which revealed MRSA bacteremia on blood culture, elevated C reactive protein, and pulmonic valve vegetation on transesophageal echocardiogram. Interestingly, white cell counts, and lactate levels were normal throughout the hospital stay. The patient had a contributing risk factor of a recent glucocorticoid injection leading to sepsis and septic shock. The diagnosis was particularly made based on the clinical features of fluid refractory hypotension, fever, and signs of end-organ hypoperfusion such as delayed capillary refill.

### CONCLUSION

Clinical examination is becoming obsolete with time and clinicians are relying more on laboratory findings. This study emphasizes that a thorough bedside physical examination should always remain the cornerstone of clinical practice in managing the patient during the initial hours of critical illness and laboratory values supplement the diagnosis of sepsis and septic shock. Furthermore, early identification of septic shock and appropriate treatment within the first three hours have a positive impact on quality of life as well as morbidity and mortality.

